# The application of mental health legislation in younger children

**DOI:** 10.1192/pb.bp.114.048959

**Published:** 2015-12

**Authors:** Victoria Thomas, Barry Chipchase, Lisa Rippon, Paul McArdle

**Affiliations:** 1 Northumberland, Tyne and Wear NHS Foundation Trust

## Abstract

We review a case history of a young child who was admitted to an in-patient mental health unit due to extremely challenging behaviour and review the legal issues that had to be considered in ensuring that there was appropriate legal authority for the child's admission and treatment. In this particular case, the patient was detained for assessment under section 2 of the Mental Health Act 1983. This case demonstrates that all clinicians working in this area require a good understanding of the law in relation to treatment of children with mental disorder, which is extremely complex.

## Competence, parental responsibility and zone of parental control

Much of the debate around consent to treatment by young children has focused on competence. ‘A child who has attained sufficient understanding and intelligence to be able to understand fully what is involved in the proposed intervention will be regarded as competent to consent’.^1^ This concept is known as Gillick competence.^2^

However, especially for younger children, persons with parental responsibility are, in general, responsible for treatment decisions. Parental responsibility is defined by the Children Act 1989 as ‘all the rights, duties, powers, responsibilities and authority which by law a parent of a child has in relation to the child and his property’. The Code of Practice for the Mental Health Act^†^ advises that the person responsible for the care and treatment of the patient must determine whether a person with parental responsibility has the capacity, within the meaning of the Mental Capacity Act 2005, to make a decision about the child or young person's treatment and whether the decision is within the ‘zone of parental control’.^3^ The ‘zone of parental control’ is a concept derived largely from European Court of Human Rights case law and has become central to parent-related decision-making.^3,4^

Although not a clearly defined concept,^5^ there are guidelines in the Code about whether a particular decision falls within the zone of parental control. There are two key questions.^3^ First, ‘is the decision one that a parent would be expected to make, having regard both to what is considered to be normal practice in our society and to any relevant human rights decisions made by the courts’? Second, ‘are there no indications that the parent might not act in the best interests of the child or young person’? The less confident a professional is that they can answer ‘yes’ to both questions, the more likely it will be that the decision falls outside the zone.

### The zone of parental control and deprivation of liberty

The Code acknowledges that the parameters of the zone will vary from one case to the next, but the following factors should be considered: the nature and invasiveness of what is to be done to the patient (including the extent to which their liberty will be curtailed); whether the patient is resisting; the general social standards in force at the time concerning the sort of decisions it is acceptable for parents to make; the age, maturity and understanding of the child or young person; and the extent to which a parent's interests may conflict with those of the child or young person. Certain treatments that could be considered particularly invasive or controversial, for example electroconvulsive therapy (ECT), are likely to be considered to fall outside the zone of parental control.^3^

Decisions that would result in a deprivation of liberty will be outside the zone, as detention engages the article 5 rights of the child (Convention for the Protection of Human Rights and Fundamental Freedoms right to liberty and security) and a parent may not lawfully detain or authorise the detention of a child.^6^ There is no specific definition of deprivation of liberty, but various factors have been identified that are likely to be relevant; for example, the use of restraint (including sedation) to admit a person to an institution where that person is resisting admission; staff exercising complete and effective control over the care and movement of a person for a significant period; and the person being unable to maintain social contacts because of restrictions placed on their access to other people. A recent judgment from the Supreme Court,^7^ commonly referred to as the Cheshire West case, has provided a clear ‘acid test’ on the meaning of deprivation of liberty. The Supreme Court has made it clear that, for a person to be deprived of their liberty, they must be subject both to continuous supervision and control and not be free to leave. The Supreme Court also held that, in all cases, the following are not relevant to the application of the test: (1) the person's compliance or lack of objection; (2) the relative normality of the placement (whatever the comparison made); and (3) the reason or purpose behind a particular placement.

One of the matters the Court considered in the Cheshire West case was whether children could be deprived of their liberty in the family home. It was noted that all children are (or could be) subject to some level of restraint. The necessity for this adjusted with their maturation and change in circumstances. The Court expressed the view that ‘very young children … because of their youth and dependence on others, have – an objectively ascertainable – curtailment of their liberty but this is a condition common to all children of tender age. There is no question, therefore, of suggesting that infant children are deprived of their liberty in the normal family setting.’ In the case of children living at home with either birth or adoptive parents, Lord Neuberger said that: ‘what might otherwise be a deprivation of liberty would normally not give rise to an infringement of article 5 [of the Convention] because it will have been imposed not by the state, but by virtue of what the Strasbourg court has called “the rights of the holder of parental authority”.’ Foster placements were viewed differently because children would generally have been placed in this environment by local authorities and therefore if there was a deprivation of liberty it would be ‘imputable’ to the state.

Prior to the Cheshire West case, many clinicians viewed the level of supervision in place in a hospital environment as amounting to a restriction, rather than a deprivation, of liberty. Since the case, there have not been any reported cases specifically considering the position of children and young people in hospital. It is inevitable however that, in the wake of the judgment, a number of children and young people who lack competence or capacity to consent to their admission to hospital and who are being treated on an informal basis will need to be assessed to evaluate whether they are being deprived of their liberty.

Hence, there will be situations where neither the consent of the patient nor parental consent may be relied on and an alternative legal authority for treatment will be necessary. The following case example demonstrates how clinicians working with children and young people are now required to manage difficult clinical scenarios within a complicated legal framework. Here we describe the reasoning behind the use of the Mental Health Act 1983 in an unusually young patient. We have been unable to find a published example of use of the Act in such a young child.

## Case study

B was an eight-year-old boy admitted to a child and adolescent mental health in-patient unit as an emergency, because of extremely challenging behaviour. He had been referred to his local community child and adolescent mental health service several months previously and was diagnosed with autism spectrum disorder. He also exhibited features of hyperkinetic conduct disorder. He was subject to a child protection plan and accommodated by the local authority on a voluntary basis at the time of admission. Because of episodes of extreme unprovoked aggression and sexualised behaviour B had been excluded from a special school and two foster placements had broken down.

On admission, B received a comprehensive package of care, which included assessments and interventions by nursing and medical staff, psychologists and other therapists. He received a carefully structured intervention involving nurses experienced with younger children, play therapy, education appropriate to his developmental level and medication (methylphenidate). B was nursed away from the older adolescents within a self-contained children's area of the in-patient ward. He had two members of nursing staff with him at all times because of his challenging behaviours, including highly sexualised behaviour, physical aggression and destruction of property. He required regular, difficult restraints involving up to four members of staff at a time, and occasional use of seclusion to maintain his own safety and the safety of others.The Code of Practice for the Mental Health Act advises that seclusion of an informal patient should be taken as an indication of the need to consider formal detention.^3^

With legal advice from trust and local authority solicitors, it was agreed that as long as B met criteria for detention under the Mental Health Act 1983, this was the preferred route. The ‘least restriction’ principle of the Act suggests that detention under the Act should be the last resort. However, it is undoubtedly necessary in cases where the option for informal admission is not appropriate or the risks in managing the child informally are too great. A patient may be detained under section 2 of the Mental Health Act 1983 for a period of assessment of up to 28 days. The application is based on the recommendations of two medical doctors, and an approved mental health practitioner is the applicant. The professionals must be satisfied that the following grounds are met:

- the person is suffering from a mental disorder of a nature or degree which warrants their detention in hospital for assessment (or for assessment followed by treatment) for at least a limited period; and
- the person ought to be so detained in the interests of their own health or safety or with a view to the protection of others (para. 4.2).^3^

In this case, B both had mental disorder (autism spectrum disorder and hyperkinetic conduct disorder) and was presenting in a way that put his own safety, and that of others, at risk.

Following a Mental Health Act assessment and close consultation with local authority and trust legal services, B was detained under section 2. B appealed to the mental health tribunal with the assistance of his independent mental health advocate and solicitor. His detention was upheld. During the period of detention, the local authority obtained an interim care order and acquired parental responsibility. The local authority questioned whether it would be able to agree to B being in hospital informally, however, the clinical team felt that the treatment decisions about restraint and seclusion required fell outside of the zone of parental control, regardless of who had parental responsibility. B's behaviours did begin to settle and he gradually ceased to require the restraint and seclusion that he had earlier in his admission. He was therefore discharged from section 2 shortly before the end of the 28-day period and remained on the ward as an informal patient while an appropriate community placement could be identified. Following several months' intervention it was possible to discharge B safely to a children's home, where he has not required restraint.

## Discussion

Detention of such a young child using the Mental Health Act 1983 is unusual and we could find no published case that would discuss this, although, anecdotally, others have faced similar decisions.

In this case, the team was confident that B's age and immaturity prevented him from being regarded as Gillick competent and therefore he could not provide authority for his own admission and treatment. Both of B's parents had parental responsibility and were supportive of his admission to hospital. Initially, the clinical team had relied on their agreement. However, in the light of B's deprivation of liberty parental consent to treat him could not be relied upon. In addition, the child protection plan raised concerns about the parents' ability to act in the best interests of the child. The team therefore decided that the decisions that now needed to be made about B fell outside of the zone of parental control.

In emergency situations, a doctor can lawfully treat a child even if there is no time to obtain valid consent.This is known as the doctrine of necessity. The Code of Practice for the Mental Health Act advises that: ‘In such cases, the courts have stated that doubt should be resolved in favour of the preservation of life, and it will be acceptable to undertake treatment to preserve life or prevent irreversible serious deterioration of the patient's condition’ (para. 36.51).^3^ In B's case, incidents of extremely challenging behaviour required urgent intervention, but these were frequent and repetitive and therefore the clinical team was unable to rely on the doctrine of necessity.

If a child is subject to a care order or emergency protection order under the Children Act 1989, the local authority acquires parental responsibility (Children Act 1989 s 33(3)(a) and s 44(4)(c), respectively). Section 25 of the Children Act 1989 can be used to detain a person with mental disorder under a secure accommodation order, but only if the primary purpose of detention is not to provide treatment for mental disorder, for example, if detention is required to maintain the safety of someone who exhibits severe behavioural disturbance. A child subject to a section 25 order does not have to be subject to an interim care order. The Children Act 1989 does not, however, specifically address mental disorder, does not provide specific powers to enforce treatment, and does not provide specific safeguards for the rights of the detained patient.^8^ B needed to be in hospital for further treatment of his mental disorder and therefore a secure accommodation order was not judged appropriate at that time.

In some situations, particularly if there are disputes between the family and the treating clinician or between family members, or if other authorities for treatment are not appropriate, there should be recourse to the courts. The High Court can use its inherent jurisdiction to make decisions that it considers to be in the child's best interests. Some issues may be resolved by section 8 orders made under the Children Act 1989. B met the criteria for detention under section 2 of the Mental Health Act and therefore the legal authority for B's assessment and treatment was provided without a court application needing to be made.

Detention under the Mental Health Act 1983 provides the child with a number of important safeguards, such as the right to appeal against detention. The 2007 amendments to the Act have resulted in greater protections for the rights of children and young people, for example the duty to ensure an age-appropriate environment (s 131A) and further safeguards for ECT (s 58A).

It is important that clinicians working with children with mental disorders equip themselves with a good understanding of the law and its application, in order that the appropriate legal authority for admission and treatment is used, taking into account all of the needs of the patient and the relevant factors of each case. The Mental Health Act 1983 can be appropriately applied to children, as this case illustrates.

## Footnotes

† This article was completed before the publication of the new Code of Practice for the Mental Health Act 1983.
